# Low-dimensional organization of angular momentum during walking on a narrow beam

**DOI:** 10.1038/s41598-017-18142-y

**Published:** 2018-01-08

**Authors:** Enrico Chiovetto, Meghan E. Huber, Dagmar Sternad, Martin A. Giese

**Affiliations:** 1 Section for Computational Sensomotorics, Department of Cognitive Neurology, Hertie Institute for Clinical Brain Research, Centre for Integrative Neuroscience, University Clinic Tübingen, Tübingen, Germany; 2 Department of Mechanical Engineering, Massachusetts Institute of Technology, Cambridge, Massachusetts, USA; 3 Departments of Biology, Electrical and Computer Engineering, Physics, and Physical Therapy, Movement Science and Rehabilitation, Northeastern University, Boston, Massachusetts, USA

## Abstract

Walking on a beam is a challenging motor skill that requires the regulation of upright balance and stability. The difficulty in beam walking results from the reduced base of support compared to that afforded by flat ground. One strategy to maintain stability and hence avoid falling off the beam is to rotate the limb segments to control the body’s angular momentum. The aim of this study was to examine the coordination of the angular momentum variations during beam walking. We recorded movement kinematics of participants walking on a narrow beam and computed the angular momentum contributions of the body segments with respect to three different axes. Results showed that, despite considerable variability in the movement kinematics, the angular momentum was characterized by a low-dimensional organization based on a small number of segmental coordination patterns. When the angular momentum was computed with respect to the beam axis, the largest fraction of its variation was accounted for by the trunk segment. This simple organization was robust and invariant across all participants. These findings support the hypothesis that control strategies for complex balancing tasks might be easier to understand by investigating angular momentum instead of the segmental kinematics.

## Introduction

Walking on a narrow beam is a demanding motor skill that requires the control of dynamic stability, defined as the ability to reduce self-initiated or external perturbations via inherent restoring moments to avoid loss of balance. Due to the reduced base of support of the beam and the intrinsic variability of the human, walking tends to become unstable in the medio-lateral (ML) direction^1^. Walking on a beam has been investigated in multiple studies over the last two decades. For instance, several studies examined the effects of age on balance control^2^, the effects of physical guidance on motor learning^3^ and the neural activation associated with loss of balance control^4^. More recently, Sawers and colleagues used beam walking as an experimental paradigm to investigate how long-term training affects muscle synergies^5^ and how individual differences in proficiency may inform therapists about clinical problems^6^. It is important to note that in all these studies, participants were asked to fold their arms in front of the body to isolate “locomotor balance” from the complex arm movements typically employed to assist balance control.

Hof suggested that for a standing human only 3 mechanisms are available for the control of dynamic balance^7,8^: (1) the shift of the center-of-pressure under the feet with respect to the vertical projection of the center-of-mass (COM), (2) the rotation of the body segments to counterbalance the variations of the angular momentum (AM), and (3) the application of external forces. The first strategy has been usually referred to also as “ankle strategy”^9^, while the second one comprises the “hip strategy”^9^, i.e. rotation of the upper body segments around the ankle or hip joint, respectively. These mechanisms can also be exploited to maintain balance in the ML direction when walking on a narrow beam, although shifting the center-of-pressure under the feet becomes a relatively ineffective balancing strategy, as the beam has a limited width. If holding onto an external object to apply an external force is not possible, rotating the body segments to create compensatory angular momenta remains the only effective strategy. A systematic investigation of the angular momentum during beam walking thus might provide deeper insights into the control strategies in such challenging balancing tasks.

The angular momentum is a physical quantity that characterizes the rotational inertia of an object or a system of objects about an axis. In any inertial reference frame, the AM of a system is a conserved quantity as long as no external forces or torques act on the object. This is the consequence of Euler’s dynamic laws of motion, according to which the derivative of AM is equal to the external torques applied to the body^10^. During walking, however, ground reaction forces are constantly acting on the feet, inducing considerable variations of the AM that need to be controlled^11^. Multiple studies in biomechanics and motor control have examined the AM to characterize the mechanisms underlying locomotion and balance control^11–15^. These studies have inspired the design of robust motion generation policies for robots and computer graphics applications^16–19^. Importantly, the AM is computed with respect to an axis about which the object rotates in space, rendering it a relative measure. In biomechanical studies, it has been common practice to compute the AM with respect to the axis passing through the whole-body center-of-mass. However, given the complex dynamics of articulated limbs when walking on a beam, it is not a priori guaranteed that this axis is the only or most appropriate choice.

The aim of this study was to investigate the coordination of a complex whole-body movement such as walking on a narrow beam. We collected movement kinematics of 16 healthy participants that were asked to complete 20 successful walks on a very narrow beam placed on the floor. Analyzing the rotations of the body segments projected into the medio-lateral plane, the segmental AM contributions were computed with respect to 3 different axes: (1) the axis perpendicular to the ML plane through the average center position of the head, (2) the axis perpendicular to the ML plane and passing through the COM of the whole body, and (3) the axis perpendicular to the ML plane through the center of the beam on the floor. The choice of the axis through the head was motivated by previous studies that showed that stabilization of the head might be an important control principle for many locomotion tasks^20^. The long axis of the beam was chosen because the human body can be seen as an inverted pendulum that rotates about this axis. The analyses of the AM components were confined to rotations in the ML plane with respect to those axes parallel to the walking direction.

Analyses of kinematic variations revealed a very complex structure with large inter-individual differences and no apparent invariances. In contrast, analyses of the AM with respect to the axis along the beam rendered a very parsimonious description of the observed coordination patterns showing a low-dimensional structure of the AM. These findings suggest that the underlying control strategy might aim at minimizing the variation of the AM about this axis.

## Results

**Figure 1:**
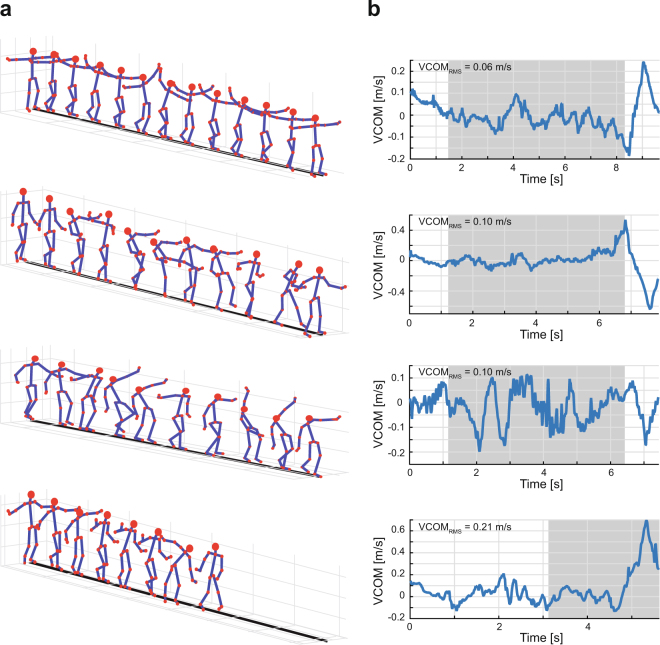
(**a**) Stick figures with segmental orientations reconstructed from 3D kinematic data of 4 representative participants walking across the beam. The first 3 panels illustrate typical successful trials and the variety of body postures adopted by different participants. The bottom panel shows the body postures seen during one unsuccessful trial. (**b**) Time series of the medio-lateral velocity of the participants’ whole-body center-of-mass associated with the trials in Figure 1a. The grey-shaded areas indicate the intervals over which the VCOM_RMS_ were computed.

While walking on the 3.4 cm-wide beam, participants displayed highly variable motor behavior, using a wide range of strategies in order to maintain or regain balance. As an illustration, Figure 1a displays 4 series of body postures that participants adopted during 4 typical trials; 3 were successful and one was unsuccessful and the participant had to step off the beam. Participants displayed not only large trunk movements, but also large and variable movements of both arms. When at the brink of falling off the beam, they also abducted their legs. Movies from a set of experimental trials are provided as supplementary material to this article. As one index to quantify the degree of balance, Figure 1b depicts the time series of the medio-lateral velocity of each of the participants’ whole-body center-of-mass (VCOM). The root mean square (RMS) of this variable was computed over 15% to 85% of the duration of each successful trial or over the last 3 seconds prior to loosing balance. The interval for this computation is highlighted in grey in Figure 1b. Comparison of the VCOM_RMS_ for all successful and unsuccessful trials confirmed that the variations in successful trials were significantly smaller, VCOM_RMS_ = 0.03 ± 0.01 m/s, than in those trials when participants lost their balance, VCOM_RMS_ = 0.15 ± 0.08 m/s, t_139_ = −170.6, p < 0.001.

**Figure 2:**
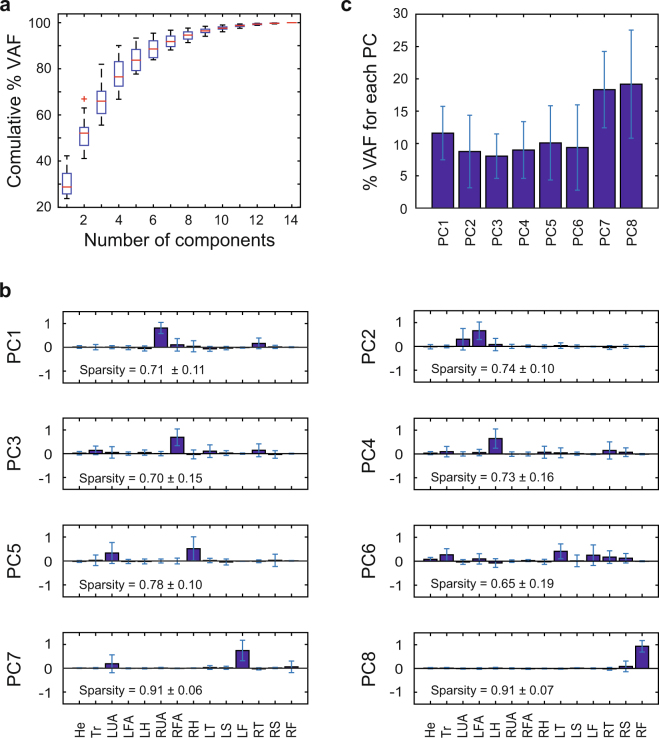
(**a**) Boxplots of the average percentage of VAF as function of the number of principal components identified from covariance matrix associated with the joint angles. (**b**) Average values of the first 8 kinematic principal components after VARIMAX rotation. Each element of the components corresponds to a specific body segment among the following ones: head (He), trunk (Tr), left and right upper arms (respectively LUA and RUA), forearms (LFA, RFA), hands (LA, RA), thighs (LT, RT), shanks (LS, RS) and feet (LF, RF). (**c**) Average percentages of variance accounted for of the 8 principal components. In all panels, average values were computed across 16 participants and the error bars represent one standard deviation.

The highly variable behavior was also evident in a principal component analysis (PCA) applied to the relative orientations of the segments. Figure 2a shows the variance accounted for (VAF) for the successful trials as function of the number of principal components, averaged across 16 participants. The whole-body movements required on average 8 components to account for at least 95% of the variance. After applying a VARIMAX rotation, we obtained components with average sparsity indexes ranging between 0.65 and 0.91 (Fig. 2b). By definition, the sparsity index is 1 if only one single element of the vector is different from 0, when more components are non-zero then sparsity is lower than 1 (see Methods for more detail). All components accounted for similar amounts of variance (Fig. 2c).

In order to test whether participants have improved over the 20 trials and changed their strategy, we split the 20 trials in 2 blocks: block 1 comprised the first 10 successful trials, block 2 the second 10 successful trials. The same PCA was applied to the covariance matrices associated with the 2 blocks. We found that for both blocks 8 components were needed to account for 95% of the variation associated with the data. Comparing the components of the 2 blocks revealed that they were similar, *S* = 0.90 ± 0.14. This average similarity index *S* was quantified as the dot product between 2 components, normalized with respect to their norms. By definition, the index *S* is equal to 1 when the components are proportional (see Methods for details). These results suggested that subjects’ strategy did not change significantly across the duration of the experiment, i.e., there was no sign of learning effects.

In order to assess the inter-individual variations of the kinematic coordination structure, we applied a cross-validation procedure. For this purpose, the principal components of a single participant were used to predict the data from each of the other participants. The amount of variance explained for all pairwise comparisons was low, the average VAF was 44.05 ± 26.45%, indicating little consistency between the kinematic strategies of the individual participants.

**Figure 3:**
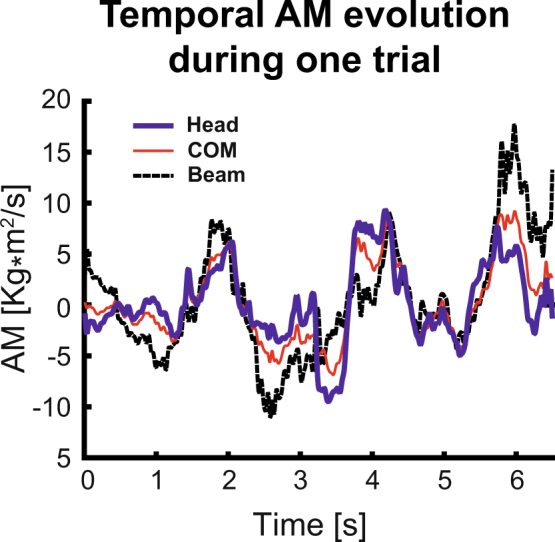
Temporal evolutions of the whole-body angular momentum during the first trial illustrated in Figure 2. The 3 lines refer to the axis through the head, the center-of-mass of the whole body, and the beam.

Given this high dimensionality and large inter-individual differences in the kinematics, we proceeded to calculate the angular momenta of the body segments. While the typical axis used for this calculation is the axis through the whole-body COM, we also calculated AM with respect to 2 additional axes, one parallel to and through the beam and one through the central position of the head. Figure 3 shows the temporal evolution of the angular momentum through the 3 axes from the trial in the top panel of Figure 1. While the 3 time series are visibly correlated, they also display considerable differences in amplitude and variability. These qualitative observations were confirmed by the average correlation coefficients (*R*) and RMS values (L_RMS_) associated with the angular momenta, which are summarized in Table 1.

**Table 1:** Average correlation coefficients (*R*) and RMS values (L_RMS_) associated with the angular momenta computed with respect to the 3 axes.

Measure	mean ± sd
*R*_*Head-COM*_	0.95 ± 0.03
*R*_*COM-Beam*_	0.91 ± 0.05
*R*_*Head-Beam*_	0.76 ± 0.11
L_RMS_(Head) [Kg m^2^/s]	1.44 ± 0.61
L_RMS_(COM) [Kg m^2^/s]	1.54 ± 0.62
L_RMS_(Beam) [Kg m^2^/s]	1.97 ± 0.76

The RMS values were computed considering only the AM components parallel to the walking direction and causing rotation along the ML plane. Like the correlation coefficients, were averaged across trials and participants.

**Figure 4:**
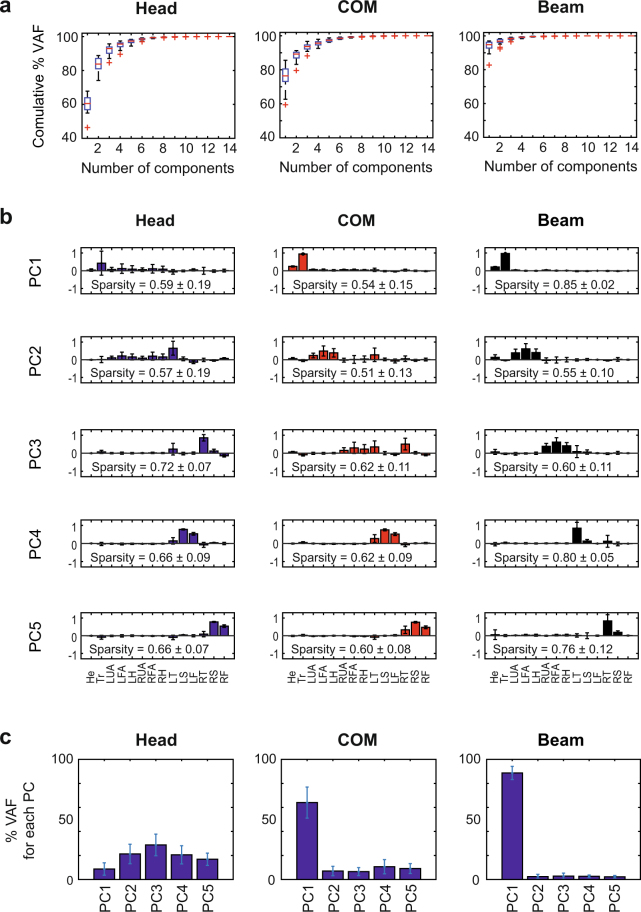
(**a**) Box-plots of the average percentage of VAF as function of the principal components identified from the segmental AM contributions computed about head, whole-body COM and the beam. (**b**) Average values of the first 5 principal components after VARIMAX rotation. (**c**) Average percentages of variance accounted for by these 5 principal components. In all panels, average values were computed across all participants and error bars represent standard deviations across participants.

Applying principal component analysis to the AM computed with respect to the different axes revealed substantial differences between the segmental coordination patterns. Figure 4a shows the cumulative variance accounted for each of the 3 axes. Before averaging, the principal components associated with different participants were paired and grouped according to their similarity. While 4 or 5 components were required to account for about 95% VAF for the whole-body COM and the head, the center of the beam as reference axis needed only one single component for a comparable VAF.

Figure 4b depicts the first 5 components for the 3 axes after a VARIMAX rotation. There were considerable differences between the 3 axes with respect to the structure of the extracted components. However, the axis through the beam center showed a particularly simple structure: the first PC was associated mainly with the trunk segment, the second and the third PCs with the left and right arm, and the fourth and the fifth PCs with the left and right leg. The similarity index *S* between the components identified with respect to the axis through the head and through the COM was *S* = 0.80 ± 0.10. Similarly, for the components computed with respect to the head axis and the beam axis, *S* = 0.60 ± 0.03. For the sets of components associated with the whole-body COM axis and the beam axis, *S = *0.63 ± 0.07. These moderate values of similarity give evidence that the segmental patterns of covariation were highly dependent on the reference axis chosen for the computation of the AM. Further, the analysis of the sparseness of the components revealed that the PCs computed with the beam axis were on average sparser than the corresponding PCs for the other 2 reference axes. This implies that using the beam center as axis rendered the lowest-dimensional description of the underlying signal space.

Figure 4c illustrates how the variance was distributed across the different components. When AM was computed with respect to the head axis, all components accounted for a comparable amount of data variation, with PC1 accounting for the smallest amount. In contrast, using the COM or the beam as axis, the biggest contribution to data variation came from the first component alone, leaving only a small amount of variance to be explained by the other components. For the beam axis, PC1 accounted for approximately 90% of the variance. Moreover, for the latter case, the average similarity of PC1 across different participants was high, *S* = 0.99 ± 0.01. This indicates that the component accounting for the majority of the variance was also relatively invariant across participants.

To test whether these results changed across practice, the data were again split into 2 blocks (first 10 and second 10 of the successful trials) and PCA was applied separately to each block. There were no noteworthy differences between the identified components, suggesting that there were no performance improvements during the experiment. When the AM was computed with respect to the head axis *S* was 0.93 ± 0.11 between block 1 and block 2. When the AM was computed with respect to the COM, *S* was 0.94 ± 0.13, and when the center of the beam was taken as reference axis *S* was 0.99 ± 0.02. The amount of variance accounted for by the first 5 PCs was always higher than 95%.

As with the kinematic results, we also quantified the reproducibility of the coordination structure between participants with a cross-validation procedure (see Methods). Using the components of a single participant, we predicted the data from other participants. With all pairwise comparisons conducted for each of the 3 axes, the reproducibility measure was substantially lower for the whole-body COM reference axis, VAF = 86.67 ± 9.77%, and for the head axis, VAF = 75.44 ± 23.80%, than for the beam axis, VAF = 98.93 ± 0.92%. These results show that the differences between individuals were much attenuated and again confirmed the robustness of the AM patterns with respect to the beam axis.

**Figure 5:**
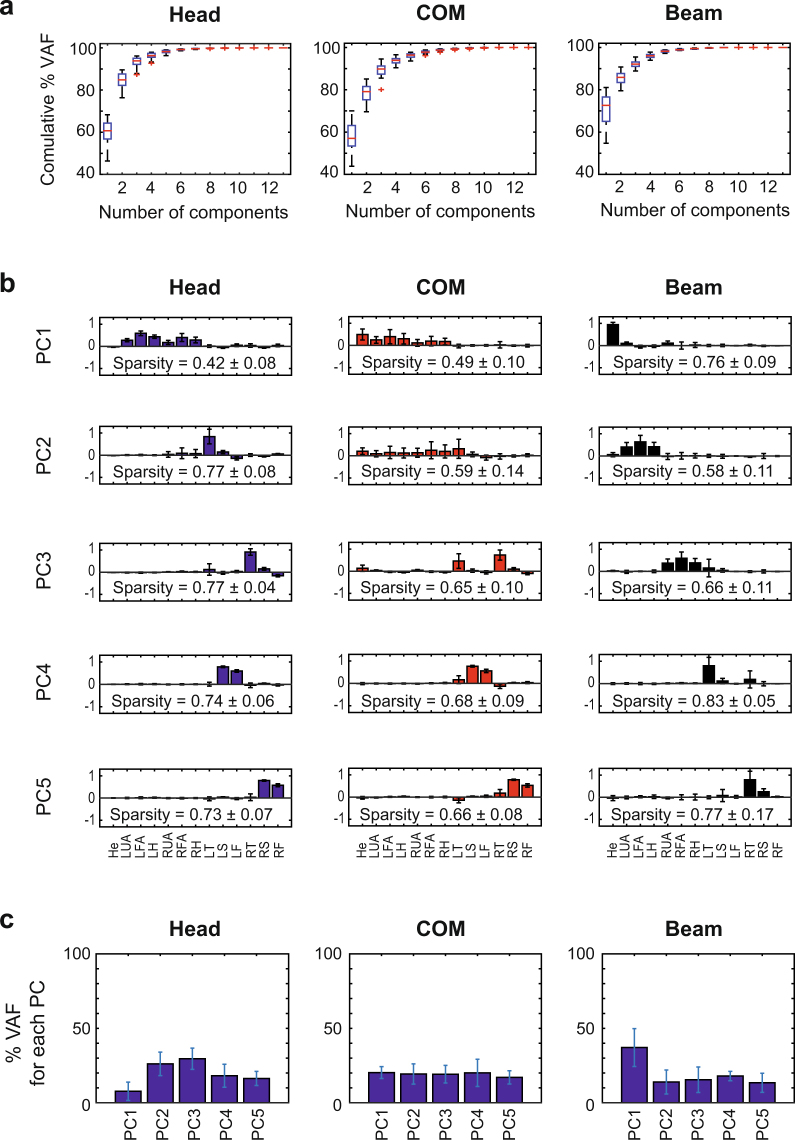
Principal component analysis of the AM contributions excluding the trunk segment. (**a**) Boxplots of the average percentage of VAF as function of the number of principal components identified from the segmental AM contributions computed about head, whole-body COM, and the beam. (**b**) Average shape of the first 5 principal components after VARIMAX rotation. (**c**) Average percentages of variance accounted for of the 5 principal components. In all panels, average values were computed across all participants and error bars represent standard deviations across participants.

One might argue that the single dominant contribution to the AM from the trunk is a trivial consequence of the fact that the trunk is the body segment with the largest mass. This might obscure the structure of the more complex coordination patterns of the arms and the legs. To evaluate this objection, we applied PCA to a reduced data set that included all AM contributions, except the one of the trunk segment. The results of this analysis are summarized in Figure 5. Figure 5a shows that even in this case 5 components explained about 95% of the variance, separately for each axis. The amount of variance that could be accounted for using one single component was however notably smaller in Figure 5a than in Figure 4a. For instance, 3 components, instead of one, were needed to account for 90% of VAF when the AM was computed the beam axis. Figure 4b depicts the first 5 principal components for the 3 axes. As above, the sets of principal components of different participants were paired and grouped according to their similarity before their averages were computed. There were still considerable differences between the 3 axes with respect to the structure of the identified components. Remarkably, the components relative to the beam axis retained their particularly intuitive structure, similar to what was seen in Figure 4b. Now, however, PC1 was associated mainly with the head segment, whereas the other 4 components were associated with the 2 arms and the 2 legs (Fig. 5b). Figure 5c illustrates how the variance was distributed across the different components. Similar to the full set of components in Figure 4c, when the AM was computed with respect to the head axis, all components accounted for a comparable amount of data variation. For the COM and the beam axis, however, the amount of VAF associated with PC1 was much lower than in Figure 4c and is comparable to the VAF associated with the other components. These results therefore support the conclusion that, even when the trunk was excluded from the analysis, the AM organization associated with the beam axis was still revealed simpler coordination patterns. This suggests that the simple AM organization was not the trivial consequence of the large mass of the trunk.

In order to assess the contributions of specific body segments to the coordination of the AM we quantified the percentage of VAF by head, trunk, arms and legs separately. The results are summarized in Table 2. Clearly, the legs alone accounted for the largest amount of variance when the AM was computed with respect to the head axis. This percentage was substantially lower for the whole-body COM and the beam axis. In contrast, the trunk segment accounted for the largest percentage of the VAF when the AM was computed with respect to the beam axis. Its contribution was smaller for the other 2 reference axes. The arms provided similar contributions when the AM was computed with respect to the head or the COM axes, but their contribution was smaller when computed with respect to the beam axis.

**Table 2:** Percentages of variance accounted for (VAF, mean ± sd) relative to 4 separate body segments, respectively head, trunk, arms and legs.

Axes	% VAF Head	% VAF Trunk	% VAF Arms	% VAF Legs
Head	0.82 ± 1.38	2.41 ± 4.05	8.07 ± 9.74	79.65 ± 13.89
COM	3.60 ± 2.57	49.37 ± 13.30	11.53 ± 10.59	27.10 ± 11.36
Beam	4.52 ± 3.52	85.34 ± 6.61	0.57 ± 4.60	1.97 ± 3.82

Besides the 20 successful trials that each participant accomplished during the experiments, we also analyzed the sets of unsuccessful trials during which participants lost their balance before arriving at the end of the beam. More specifically, for each participant we applied the same PCA with VARIMAX rotation to the segmental orientations and computed the AM contributions with respect to the beam axis. The analysis was restricted to the last 3 seconds prior to termination, defined as the moment when one foot touched the ground. The principal components of the unsuccessful trials were then compared to those of the successful ones. Using the same cross-validation procedure as above, we quantified to which extent the principal components in the successful trials could account for data variation in the unsuccessful trials. For the segmental kinematics, 8 principal components were needed to account for 95% of the variance in the unsuccessful trials, VAF = 93.93 ± 2.46%. The similarity between the components in successful and unsuccessful trials was relatively high, but was highly variable, *S* = 0.88 ± 0.21. Moreover, the principal components of the successful trials could not account for much of the variance in the unsuccessful trials, VAF = 63.22 ± 15.32%. These results suggested that when participants started to lose balance they recruited different kinematic strategies than the ones in the successful trials.

When testing the segmental AM contributions, we found that in the unsuccessful trials 5 principal components could always account for at least 99% of the variance and that these components were very similar to those associated with the successful trials, *S* = 0.95 ± 0.11. Moreover, the cross-validation procedure revealed that the principal components in the successful trials reconstructed the AM variation in the unsuccessful trials with high approximation, VAF = 98.75 ± 0.71%. These results therefore suggest that while the kinematic coordination deviated from the steady-state pattern, the loss of balance was not preceded by a recruitment of altered AM components.

The low-dimensional AM organization that characterized both successful and unsuccessful trials suggests that the AM pattern arose from a linear combination of invariant AM patterns (the PCs). To further examine this conjecture, the linear weights associated with the PCs were analyzed. The specific hypothesis was that loss of balance was brought about by a different combination of the principal components in comparison to the successful trials. To investigate this hypothesis, the coefficient of the first AM principal component (PC1), explaining at least 90% of the variance, was related to a task variable that characterized the degree of balance. The task variable that quantified ML balance, the ML velocity of the center-of-mass (VCOM) was chosen (Fig. 1b). Differences in the coefficients of successful and unsuccessful trials should reflect different recruitment processes of the component.

**Figure 6:**
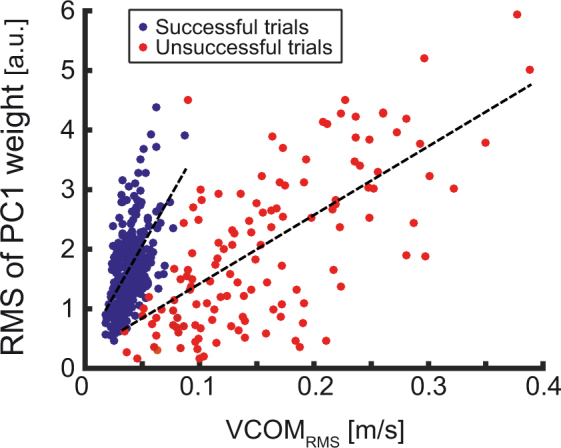
Linear regression of the RMS values of the coefficients associated with the first AM principal component against the RMS values ML velocity of the center-of-mass, VCOM_RMS_. The blue symbols denote the successful trials, the red symbols denote the unsuccessful trials. The slopes of the 2 linear regressions are visibly different.

More specifically, we hypothesized that the RMS value of the time series of the linear weight of PC1, computed within each single trial, should be predicted by the RMS of the time series of the VCOM, VCOM_RMS_, split by the type of trial (successful/unsuccessful) and their interaction. A multiple regression revealed that such a linear model predicted a significant amount of the variance associated with the weight, F_3,454_ = 118.03, p < 0.002, R^2^ = 0.44, R^2^_adju_ = 0.43. Further, the type of trials did not significantly predict the coefficient, β = −0.112, t_457_ = −0.589, p > 0.05. In contrast, both VCOM_RMS_ (β = 56.66, t_457_ = 8.06, p < 0.001) and the interaction between trial type and VCOM_RMS_ (β = −22.51, t_457_ = −6.30, p < 0.001) were significant predictors. The β values indicate the slopes associated with the corresponding predictor in the regression equation. Taken together, the regression results showed that the weight of PC1 was approximately 2 times larger in the successful trials than in the unsuccessful ones (see Fig. 6). This implies a weaker recruitment of this component in the instants prior to balance loss compared to the recruitment of the same component during the successful trials.

## Discussion

The overall goal of this study was to investigate possible control strategies underlying the observed coordination during a challenging balancing task, walking on a narrow beam. We found highly variable and complex kinematic patterns with large-amplitude movements of the trunk, arms and legs. This high variability in the kinematic patterns was reflected in the high dimensionality of the relative orientations of the segments as quantified by a PCA. In addition, there appeared to be little or no consistency of patterns across the different individuals, indicating a lack of invariance underlying the organization of the movements. Contrasting with this result, the analysis of the AM revealed a much simpler pattern. Applying PCA to the AM structure of the different body segments, we were able to find a low-dimensional and coherent structure. This was especially the case when the AM was computed with respect to an axis aligned with the beam. In contrast, when the AM was computed using the axes through the whole-body COM or the head, the revealed structures required more components to approximate the data.

Beam walking has been investigated in multiple previous studies^2–4,6^. However, in all these experiments, participants walked on the beam with their arms folded in front of the body. The implicit or explicit reason was to isolate the locomotor task from disturbances arising from the complex and irregular arm movements. Simplifying the analysis of the balancing components by excluding the influence of the arms can be advantageous when pursuing clinical goals^6^. However, it remains an open question how humans might control their entire body including their arms to regulate balance. To answer this question, we opted to not impose constraints on the arm movements and allowed maximal freedom on the choice of motor strategies to maintain balance. Indeed, participants moved their arms extensively (as visible by the loadings in PC1 to PC7 in Fig. 2), suggesting that the arms likely played a role in the control of balance. This conclusion was also corroborated by the finding that 2 out of the 5 AM components were related to the arms. This underscores that to understand the control of locomotor balance under relatively unstable conditions, it is necessary to examine arm movements.

How can these results shed light on the control of balance? Our results might be interpreted with a two-layered control framework^21,22^: an “execution” level and a “task” level. Rotating the body segments to control the whole-body AM about the beam axis to minimize the risk of falling may reflect a control strategy that is concerned with lower-level variables, such as segmental AM contributions. This view is supported by the simple AM structure: the trunk, as a segment with comparably high mass (about 43% of the total body weight^23^) and located relatively far away from the axis dominates the decomposition of the AM. The 2 legs and the 2 arms, which have much smaller mass, are components that can be used for finer control or as measures of “last resort” to avoid loss of balance. This interpretation is supported by the fact that the the RMS values of PC weights associated with the upper- and lower-limb components increased during the unsuccessful trials before participants lost balance and stepped off the beam (see supplementary Fig. S1). Note that the analysis that eliminated the trunk component ruled out that all structure was generated by the trunk component.

At the task level the human body might be approximated by an inverted pendulum that rotates about the axis that is defined by the contact of the feet with the beam^24,25^. Therefore, the corrections at the execution level might aim to ensure the dynamic stability of the inverted pendulum. This raises the question how the body is controlled to assemble and regulate this inverted pendulum? Note that an inverted pendulum is by definition unstable. Therefore, there has to be at least one additional degree of freedom to afford stability: either a joint at the hip to form a double-pendulum, or a joint at the tip of the pendulum connected like a T-bar. Interestingly, these 2-DOF linkages map into well-known strategies when balancing: a two-DOF system may be achieved by moving around the hip joint, the so-called hip strategy, well known in postural control^9^. The “T-bar model” is realized when the 2 arms are extended horizontally or even enhanced by rope walkers who hold a long horizontal bar, evidently to help them maintain stability. The observation that the left and right arms are indeed the second and third PC is consistent with this T-bar model.

The hypothesis that at the task level the whole-body system may be approximated as an inverted pendulum does not contradict the fact that, at the execution level, a complex motor strategy may be applied. The approach to approximate and simplify the whole body at the task level has revealed to also be useful in the control of robotic systems^26,27^. While balance of an inverted pendulum can be achieved by applying, for example, zero-moment-point control, more refined control requires more accurate models^28–30^.

Analysis of the unsuccessful trials provided interesting insights into the strategies that participants recruited in the last 3 seconds before losing balance. Regardless of the chosen axis used for the AM computation, the AM components recruited in this interval were similar to the ones recruited in the successful trials. On the one hand, this suggests that losing balance cannot be attributed to the recruitment of a “wrong” set of components. Instead, the analysis suggests that the “right” components may be not be recruited properly, i.e. with the wrong linear weights, to assure balance recovery. The linear regression analysis seemed to support this hypothesis. In the unsuccessful trials, the activation of the most important component (PC1) was much lower than its activation during the successful trials, given a specific amount of dynamic instability VCOM_RMS_. This can be interpreted as a decrease of control effort and one possible cause of the loss of balance. However, the experimental procedures were designed for the successful trials and more work is needed to develop more suitable procedures to reveal how the AM components are recruited and whether a deficient recruitment process can lead to loss of balance.

Angular momentum during walking has been investigated as a possible diagnostic measure for individuals with movement deficits caused, for instance, by a stroke^31–37^. Analysis of gait stability in terms of the structure of angular momentum around different axes may inform clinicians about individual deficits and may point to novel rehabilitation protocols for patients with balance problems. Our results showed that the RMS value of the medio-lateral velocity of the COM and the scalar coefficient of the first principal component can discriminate between successful and unsuccessful trials. Hence, the coefficients of the AM principal components may be informative for the assessment of balance problems and motor recovery during rehabilitation. Similarly, the number and amount of variance of principal components might help discriminate between different pathological changes and help in the design and assessment of individual rehabilitation protocols.

While the more standard clinical measures of functional impairment, such as the velocity of the COM, are easier to obtain, the typical clinical measures tend to be global descriptors. We conjecture that the parameters of the low-dimensional organization might be more sensitive to specific pathological factors and ultimately more precise and specific as diagnostic tools.

Our study showed that computing the AM with respect to axes different from the typically used axis of the whole-body COM provide novel and interpretable results. An interesting question therefore arises whether these methods and results generalize to other walking conditions. On the one hand, normal walking on flat ground is comparatively stable in the ML plane and analysis of the AM with different axes may not provide new information as the limb rotations in the ML plane are comparatively small. For example, the computation of the AM along the sagittal plane might provide useful insights as walking implies a rotational motion about the ankle of the standing leg in the sagittal plane. Similar considerations apply to walking on stairs or slopes^15,16^. On the other hand, analysis with respect to the reference axis of the head may prove insightful for many other balance-challenging walking conditions as head stabilization is an important reference for control during several locomotion tasks^20^. In conclusion, our results may serve as stimulus to consider alternative axes when analyzing whole-body control in locomotory tasks.

## Methods

### Participants

Sixteen healthy participants completed the experiment (11 males, 5 females, ages 27 ± 4 years, mass 70 ± 11 kg, height 1.76 ± 0.09 m). All participants were in good health and had no previous history of neuromuscular disease. The experiment conformed to the Declaration of Helsinki and written informed consent was obtained from all participants according to the protocol approved by the ethical committee at the Medical Department of the Eberhard-Karls-Universität of Tübingen, Germany. Participants appearing in the figures or in the supplementary videos provided informed consent for publication of identifying information/images in an online open-access publication.

### Kinematic Measurements

**Figure 7:**
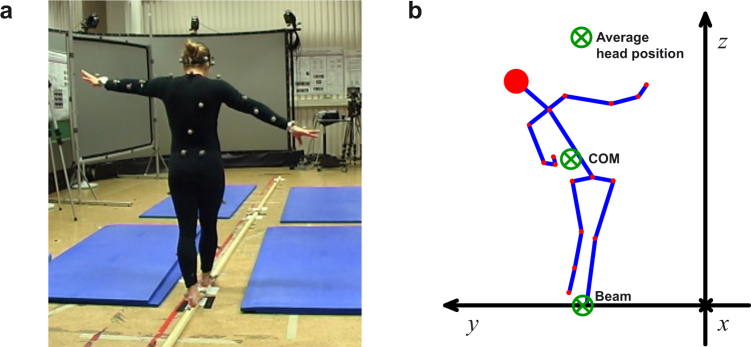
(**a**) Participant walking on the narrow beam and wearing the markers sets for 3D kinematic data acquisition. (**b**) Illustration of the projection of the joints onto the ML plane of a participant walking on the beam. The walking direction is the *x*-axis. The green circles represent the 3 axes with respect to which the AM was computed. According to equation (1), only the AM along the *x*-axis can produce rotations of the segments in the ML plane, identified by the *y*- and *z*-axis.

Kinematic data were collected with a Vicon motion capture system with 10 infrared cameras (Oxford, UK), which recorded the 3D positions of spherical reflective markers (2.5 cm diameter). The markers were attached with double-sided adhesive tape to tight clothing worn by the participants (Fig. 7a). Marker placement followed the Vicon’s PlugInGait marker set. The sampling rate was set at 100 Hz; spatial error was below 1.5 mm. To create a challenging condition for balance control participants walked on a very narrow beam (3.4 cm wide, 3.4 cm high, 4.75 m long). The beam was fixed to the ground with strong double-sided adhesive. In the Vicon frame of reference the axis parallel to the beam was defined as the *x*-axis (Fig. 7b); the axis perpendicular to the beam was defined as *y*-axis, with positive pointing leftward with respect to the direction of motion; the third axis parallel to the gravity direction was defined as *z*-axis, pointing upward.

### Experimental procedure

Each participant was asked to walk barefoot from one end of the beam to the other. Starting from a standing position with the left foot on the beam and the right foot on the ground, he/she started walking after the experimenter gave a go-signal and started the movement recording. Importantly, participants were allowed to freely move their arms to maintain balance and there were no time constraints. After reaching the end of beam, the participant stepped off the beam with both feet on either side of the beam and stood still until the movement recording was stopped. A typical successful trial lasted between 4.51 and 23.28 seconds. The participant then returned to the starting position. If the participant lost balance and stepped off the beam before reaching the end, the experimenter stopped the recording and the participant returned back to the starting position. A typical unsuccessful trial lasted between 0.96 and 18.89 seconds. Each participant performed trials until 20 successful trials were completed. After each trial, participants were allowed to take a short rest if needed. Participants needed on average 34 ± 16 trials to achieve 20 successful trials. While there were 14 unsuccessful trials per participant, this number varied widely between 0 and 52 trials across individuals.

### Data analysis

Commercial Vicon software was used to reconstruct and label the markers, to interpolate between short missing segments of the trajectories, and to compute the center-of-mass (COM) of the whole body. Kinematic analysis was performed off-line using Matlab v.R2015a (The Mathworks, Natick, MA). Before analysis, kinematic data were low-pass-filtered using a Butterworth filter with a cut-off frequency of 20 Hz. To exclude transient behaviors, only the time windows between 15% and 85% of the duration of each successful trial were considered for analysis. For each unsuccessful trial only the last 3 seconds before the participant stepped off the beam were considered. Unsuccessful trials shorter than 2.5 seconds were excluded from the analysis. After this exclusion, there were 151 unsuccessful trials in total, on average 9 ± 12 trials per participant, varying between 0 and 39 trials across individuals. As this study was mainly interested in understanding the organization of the kinematics and the AM for balance control in the medio-lateral (ML) plane, the kinematic analysis was confined to this ML plane.

#### Relative orientations of the body segments

The human body was modeled as a kinematic chain composed of 14 rigid segments: head, trunk, left and right upper arms, forearms, hands, thighs, shanks and feet. The spatial coordinates of the extrema of each segment (i.e., the ends of each link) were derived from the motion capture data. For the head, the first coordinate was obtained computing the average position between the centers of rotation of the left and right shoulder, the second coordinate was defined at the average position of the 4 markers attached to the head. These coordinates were used to determine the axis parallel to the beam but through the head. For each hand, one extremum coincided with the wrist joint, and the other one with the marker applied to the base of the index finger on the back of the hand. For each foot, the first extremum coincided with the ankle joint of rotation, the second extremum with the marker applied on the top of the big toe. The 3D spatial coordinates of the joints of rotation were projected onto the ML plane by setting the coordinates of the joints of rotation along the beam direction (*x*-axis) to zero. For each segment, its relative orientation was computed as the angle between that segment and its proximal and adjacent segment. The orientation of the trunk segment was computed with respect to the *z*-axis.

#### Angular momentum

The contribution **L**_*i*_(**r**_*p*_) of each segment to the whole-body angular momentum (AM) with respect to an axis of rotation passing through the point *P* was computed as follows:

1 $${{\bf{L}}}_{i}({{\bf{r}}}_{P})=({{\bf{r}}}_{COM,i}-{{\bf{r}}}_{P})\times {m}_{i}({{\bf{v}}}_{COM,i})+{{\bf{I}}}_{i}{{\boldsymbol{\omega }}}_{i}$$

where **r**_*COM*,*i*_ indicates the position vector of the center-of-mass of the *i*-th segment, **v**_*COM*,*i*_ its corresponding velocity, **I**_*i*_ its inertial tensor, and ω_*i*_ its corresponding 3D angular velocity. **r**_*p*_ indicates the position vector of the point *P*. For each segment, the position of the corresponding center of mass and the inertial tensors **I**_*i*_ were computed using average human anthropometric data and the kinematic measures derived from the motion capture data^38,39,23^. As the analysis focused on the movements in the ML plane, only the AM component parallel to the *x*-direction was considered. This is the component of the vector **L** that causes rotations of the body segments in the ML plane.

For each participant, the AM was computed about 3 axes passing through 3 different points in the ML plane (Fig. 7b): (1) the average position of the head computed across all trials, (2) the position of the whole-body COM over time, and (3) the center of the beam. The center of the beam and the average position of the head of each participant were fixed points in the ML plane. In contrast, the position of the whole-body COM changed over time. However, it is well known that, for the COM, the derivative of the AM computed with respect to the *x*-axis passing though the COM is always equal to the external moments applied to the body. The Euler’s law^10^ was therefore always valid independently of the chosen axis. The total AM was computed by summing the contributions of all body segments:

2 $${\bf{L}}({{\bf{r}}}_{P})={\sum }_{i=1}^{14}{{\bf{L}}}_{i}({{\bf{r}}}_{P}),$$

#### Index of stability

The velocity of the whole-body center-of-mass along the ML direction, VCOM, showed marked fluctuations in time, coincident with variations in segmental kinematics (see Fig. 1 for illustration). To characterize this ML velocity, the root mean square error (RMS) was calculated over the specified duration of each successful and unsuccessful trial VCOM_RMS_.

#### Analysis of dimensionality

Principal component analysis^40^ (PCA) is an unsupervised learning method that allows to decompose an input matrix **X** into the linear combination of a finite set of orthonormal basis vectors, referred to as principal components. These basis vectors are weighted by a set of scalar coefficients. In order to analyze the spatio-temporal coordination of the relative orientations of the body segments, PCA was applied to the covariance matrix of the segmental orientations. To analyze the dimensionality of the segmental AM contributions, PCA was applied to the covariance matrix of the AM components parallel to the walking direction, i.e. the direction causing the rotation of the body segments in the frontal plane. In order to reduce the variability across study participants, the AM contributions were represented in dimensionless form prior to PCA. For this purpose, they were normalized with respect to the product between the participant’s body mass (kg), walking speed (m/s) and body height (m). The covariance matrix was used instead of the correlation matrix to avoid any amplitude normalization of the signals from different orientations or body segments. Subsequently, only the minimum number of components was retained that was sufficient to account for at least 95% of the total variance (VAF)^41^.

A VARIMAX rotation was applied to the retained components to simplify the interpretation of the factors^42^. The direct effect of the VARIMAX rotation was a sparsification of the components, making the elements of each component very small or zero. The fewer elements of the components are different from zero, the easier it is to provide a functional interpretation of the components. To quantify the level of sparseness for each rotated factor, Hoyer’s index was used^43^. More specifically, sparsity was defined as follows:

3 $${\rm{Sparsity}}=(\sqrt{N}-{\ell }_{1}/{\ell }_{2})\cdot {(\sqrt{N}-1)}^{-1},$$

where *N* indicates the number of elements in each component (here *N = *14 for both segmental orientations and AM contributions) and $\ell$_1_ and $\ell$_2_ indicate the 1-norm and the 2-norm, respectively. This measure varies between 0 and 1, where zero means that the factor is not sparse, and 1 signifies the maximum level of sparsity, where only one element of the factor is different from zero.

#### Similarity between components

In order to assess similarity between different principal components associated with different axes, different participants, or the 2 types of trials (successful versus unsuccessful), a similarity measure *S* was computed for all possible pairings. To obtain the similarity *S*, the corresponding scalar product was computed after the components were normalized with respect to their norms. Thus, given 2 principal components **u** and **v**, their similarity was defined as follows:

4 $${S}=\frac{{\bf{u}}\cdot {\bf{v}}}{\Vert {\bf{u}}\Vert \Vert {\bf{v}}\Vert }$$

where $\Vert \cdot \Vert$ indicates the Euclidian norm. The index *S* represents the cosine of the angles between the vectors identified by the 2 components. When the index is equal to 1, the components are proportional to each other, while *S* = 0 implies that they are orthogonal. The index *S* is equivalent to the uncentered Pearson correlation coefficient.

The principal components of this first best-matching pair were then removed from the corresponding sets and the procedure was repeated for the second-best matching pair, and so forth. This procedure was iterated until all components had been matched. The computation of the similarity *S* between 2 sets of components provided a quantitative assessment of the extent to which the patterns of covariation of the segmental orientations or segmental AM contributions relative to 2 axes, 2 participants, or successful or unsuccessful trials differed from each other.

#### Cross-validation

A cross-validation procedure was performed to assess the extent to which the organizations of the relative orientations of the body segments and of the AM identified by PCA were invariant across participants or successful or unsuccessful trials. Each set of principal components identified in one participant or in one type of trial was used to reconstruct the data from the other participants by least-square approximation. The goodness of reconstruction was quantified in terms of variance accounted for. The goodness-of-fit measures were averaged across all pairings between participants to obtain a single reproducibility score. We also computed the extent to which head, trunk, arms and legs contributed separately to the total AM. Their contributions were quantified in terms of percentage of variance accounted for.

#### Statistical analysis

Mean and standard deviation were used to summarize the data. The similarity between 2 time series was quantified by computing the corresponding centered Pearson’s correlation coefficient *R*. The amplitudes of temporal signals were characterized by computing the corresponding root mean square values RMS. A Welch t-test was applied to compare RMS values of medio-lateral velocity across participants and trials, i.e. with unequal and different sample sizes. A multiple regression analysis was conducted to test whether COM-stability in the ML plane (quantified by VCOM_RMS_), the type of trial (successful/unsuccessful) and their interaction predicted the RMS value of the scaling coefficient associated with one principal component (the one associated with the trunk segment and obtained from the AM computed with respect to the trunk).

#### Data availability

The data that support the findings of this study are available from the authors of the article. Restrictions apply to the general availability of these data, which were used under license for the current study, and so are not publicly available. However, data are available from the authors upon reasonable request.

## Electronic supplementary material

Supplementary Figure

Supplementary videoS1

Supplementary videoS2

Supplementary videoS3

Supplementary videoS4

Supplementary videoS5

Supplementary videoS6
