# Author Correction: Low-dimensional organization of angular momentum during walking on a narrow beam

**DOI:** 10.1038/s41598-018-24802-4

**Published:** 2018-04-18

**Authors:** Enrico Chiovetto, Meghan E. Huber, Dagmar Sternad, Martin A. Giese

**Affiliations:** 1 Section for Computational Sensomotorics, Department of Cognitive Neurology, Hertie Institute for Clinical Brain Research, Centre for Integrative Neuroscience, University Clinic Tübingen, Tübingen, Germany; 2 Department of Mechanical Engineering, Massachusetts Institute of Technology, Cambridge, Massachusetts USA; 3 Departments of Biology, Electrical and Computer Engineering, Physics, and Physical Therapy, Movement Science and Rehabilitation, Northeastern University, Boston, Massachusetts USA

Correction to: *Scientific Reports* 10.1038/s41598-017-18142-y, published online 08 January 2018

Six supplementary movie files containing videos from experimental trials were omitted from the original version of this article. This has been corrected in the HTML version of the Article; the PDF version was correct at time of publication.

