# Supplementary material for: Low-dimensional organization of angular momentum during walking on a narrow beam
Source: Sci Rep. 2018 Jan 8;8:95. doi: 10.1038/s41598-017-18142-y (PMC5758518; doi:10.1038/s41598-017-18142-y)
Supplement: Supplementary file 1 — Supplementary Figure [file 41598_2017_18142_MOESM1_ESM.doc]

**Low-dimensional organization of angular momentum during walking on a narrow beam**

Enrico Chiovetto, Meghan E. Huber, Dagmar Sternad, Martin A. Giese


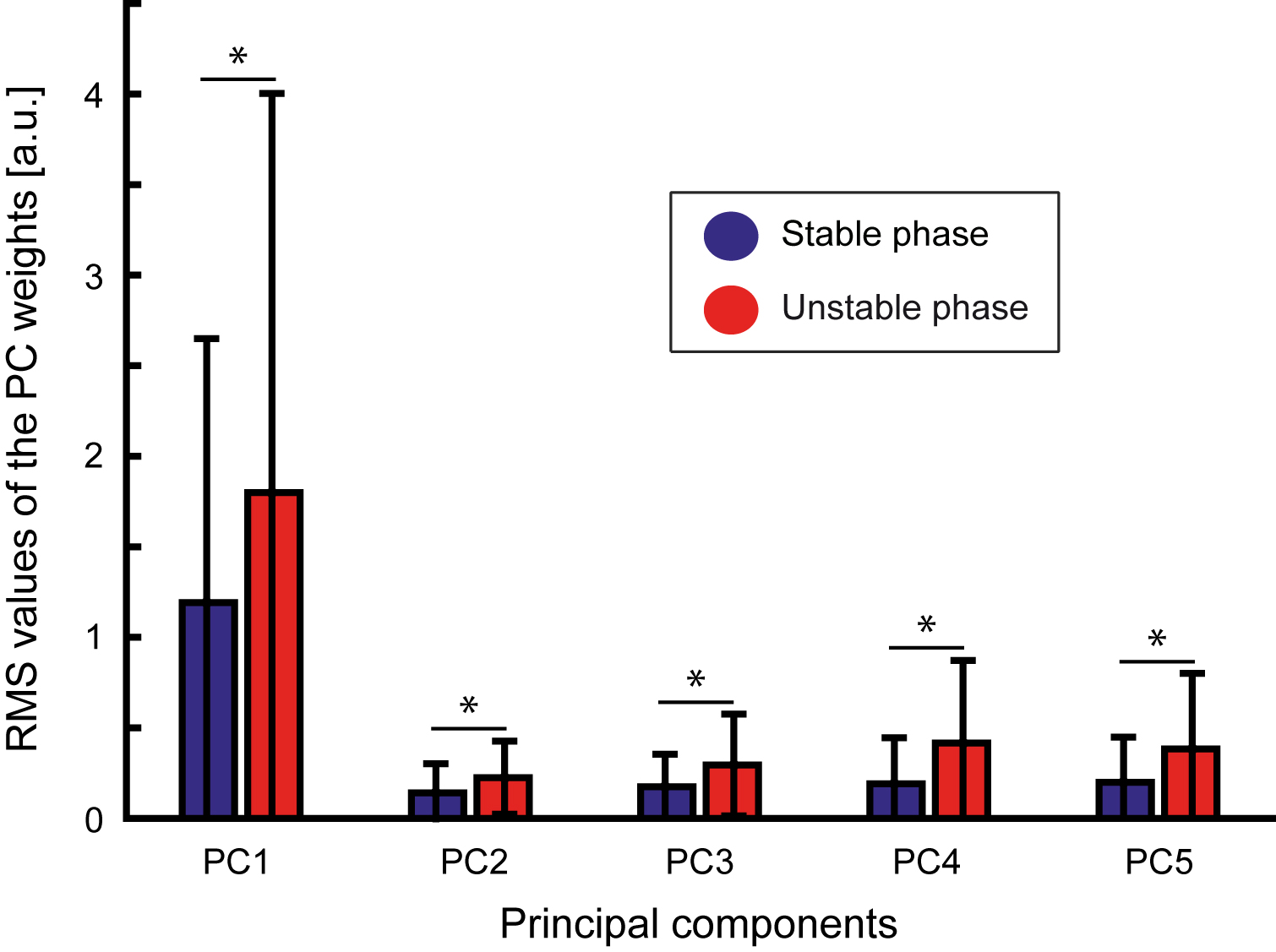


**Figure S1. RMS values of the PC weights during the stable and unstable phases of unsuccessful trials.** To assess how the PC structure changed prior to loosing balance, the RMS values of the linear weights of each PC were computed over two different intervals: the unstable phase was defined as the last 3 seconds preceding the loss of balance; the stable phase was defined as the interval preceding the unstable phase. The PC weights of the stable phase were computed by optimizing the fitting of the AM data using the PCs identified in the unstable phase. This fitting procedure rendered a reconstruction accuracy of 99.34 ± 0.11% (mean ± sd). During the unstable phase the weights were significantly larger than during the stable phase (Welch t-tests, p<0.05). This result suggests that before losing balance, participants exploited as many components as possible as a “last resort”. The asterisks indicate statistically significant differences.
